# COVID-19-Related Intestinal Ischemia in A 7-Year Old Boy

**DOI:** 10.1055/s-0042-1755721

**Published:** 2022-08-16

**Authors:** Balázs Fadgyas, Gábor István Garai, János Schnur, Viktória Irén Kiss, Viktória Vass, Enikő Mátyus, György Balázs, Tamas Cserni

**Affiliations:** 1 Department of Surgery and Traumatology, Heim Pál National Institute of Paediatrics, Budapest, Hungary; 2 Department of Anaesthesia & Intensive Care, Heim Pál National Institute of Paediatrics, Budapest, Hungary; 3 Department of Pathology, Heim Pál National Institute of Paediatrics, Budapest, Hungary; 4 Center of MRI and CT Diagnostics, Heim Pál National Institute of Paediatrics, Budapest, Hungary; 5 Department of Pediatric Surgery, Josa Andras Hospital Nyíregyháza, Teaching Hospital of Medical Health Science Centre University of Debrecen, Nyíregyháza, Hungary

**Keywords:** COVID-19, pediatric multisystem inflammatory syndromeintestinal ischemia, hypotension, Meckel's diverticulitis, child

## Abstract

Severe abdominal pain and vomiting are common symptoms in children with pediatric multisystem inflammatory syndrome (PIMS). Mesenteric lymphadenitis and aseptic peritonitis are predominantly reported in cases where acute surgical abdomen was suspected and laparotomy was performed at the early stage of the pandemic. These reports generally discouraged surgeons to perform exploration in COVID-19-related cases and medical management was prioritized. Only a few COVID-19-specific surgical cases with intestinal ischemia were published. Here, we report another case of COVID-19-related intestinal ischemia complicated with Meckel's diverticulitis in a non-immunocompromised child who clearly required surgical intervention. In our case, the combination of COVID-19-related vasculitis and low blood pressure episodes may have contributed to this severe outcome.

## Introduction

Early studies reported that young children were disproportionately spared from COVID-19 infection and those who became infected (2%) remained asymptomatic or mildly symptomatic with fever and cough. 1 2 This changed since April 2020, when children began presenting with Kawasaki-like disease, i.e., fever, gastrointestinal symptoms, and features of myocarditis. This disease was defined later as a pediatric multisystem inflammatory syndrome (PIMS) caused by post-viral inflammatory vasculopathy, involving multiple organs such as the brain, lungs, heart, kidneys, and bowels. 3 4

The gastrointestinal symptoms were very common (84%) and significantly severe that some of these children underwent laparotomy with the initial diagnosis of acute surgical abdomen. But the explorations only found mesenteric lymphadenitis and/or aseptic peritonitis rather than severe bacterial infection, intestinal obstruction, bleeding, or vascular occlusion requiring surgery. 5 Despite the clear role of the vasculopathy in PIMS, only a few cases of intestinal ischemia and necrosis were reported in the literature. 6 7 8 We report a case of PIMS-related intestinal ischemia and necrosis complicated with Meckel's diverticulitis and discuss the similarities and differences of the reported cases.

## Case Report

A 7.5-year-old boy in foster care, with known left-sided renal agenesis and developmental delay, presented with a 2-day history of high temperature, with no obvious upper respiratory symptoms to a virtual clinic with his general practitioner. General advice was given to his legal guardians regarding the fluid consumption and temperature control. Oral co-amoxiclav was prescribed. The patient's condition deteriorated over the following 24 to 48 hours and was referred to the local hospital. His main symptoms were headache, vomiting, facial petechiae; however, no abdominal pain or diarrhea was recorded. There was no history of a known COVID-19-positive contact.

On admission, he was severely dehydrated and showed meningism. His blood gas showed pH 7.37, pCO _2_ 34.1 mm Hg, pO _2_ 68.5 mm Hg. Raised inflammatory markers (C-reactive protein [CRP] 221 mg/L, white blood cells [WBC] 5.9 g/L, neutrophiles [Neu] 88%) were found in his laboratory results. His abdominal physical examination was unremarkable, and he passed normal stool the day of admission. His chest X-ray showed a right-sided pneumonia. An abdominal US described enlarged mesenteric lymph nodes.

Fluid and electrolyte resuscitation along with IV ceftriaxone were started immediately. COVID-19 swabs were taken. Twenty-four hours later, his respiratory parameters worsened; however, the O _2_ saturation was maintained with nasal O _2_ administration. On the same day, he developed an abdominal pain with increasing intensity and started vomiting again. His SARS-Cov-2 PCR test came back negative; however, his D-dimer was elevated to 4.2mg/L, along with a GGT (gamma-glutamyl transferase) of 225U/L and a serum bilirubin of 42πmol/L, lactate level was 1.74mmol/L, and troponin <5.5ng/L, respectively. His serology showed SARS-CoV-19 IgG antibodies (3.9 second c, reference <0.49 second c, Roche anti-SARS-CoV-2 N test).

At this point, PIMS was considered as working diagnosis, and the patient was transferred to the regional center and admitted to a pediatric ICU. He was referred to the pediatric surgeons for assessment of his abdominal pain. The surgical examination revealed a soft, non-distended abdomen, with moderate right iliac fossa tenderness, no guarding and weak bowel sounds. Ultrasound scan showed no dilated bowel loops and adequate bowel movements, bowel obstruction was not suspected at this stage and erect abdominal X-ray was not performed. To this point, observation was decided.

After 24 hours, CRP increased to 400mg/L, WBC to 30 g/L, Neu to 92.7%, and the D-dimer to 2118ng/mL. The serum lactate level increased only slightly to 3.01 mmol/L; however, troponin level increased significantly to 58.42ng/L. He had hypotension (MAP [main arterial pressure] 55, systolic blood pressure 80 Hgmm). On subsequent physical examination, abdominal distension and guarding in the right iliac fossa become obvious and no bowel sounds were detected. A CT scan of the chest and abdomen was performed and a right-sided pneumonia and very dilated, fluid- and gas-filled small bowel loops were found, with evidence of a small intestinal intussusception. The rectum was gasless. Considering the significant deterioration of the patient's general condition and the signs of bowel obstruction, exploration was decided.

**Fig. 1:**
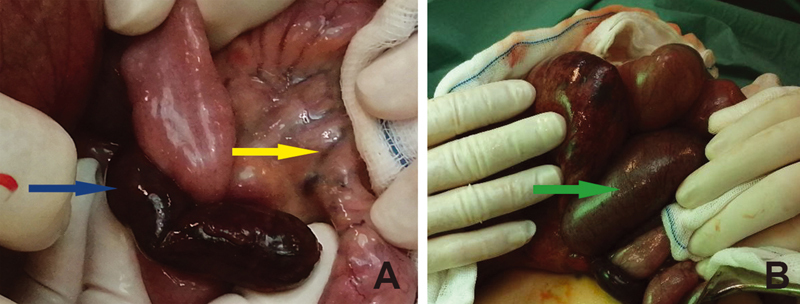
Necrotic Meckel's diverticulum obstructing the ileum (blue arrow). Thrombosis in the mesenteric vessel (yellow arrow). Dilated necrotic small intestine signed by a white arrow.

At median laparotomy, an inflamed and gangrenous Meckel's diverticulum was found, 50 cm away from the ileocecal valve, strongly attached to the adjacent ileal loop, causing obvious bowel obstruction ( Fig. 1A ). Furthermore, another 100 cm dilated small bowel proximal to the diverticulum was found necrotic and required resection ( Fig. 1B ). Thrombosis of the veins was observed in the mesentery belonging to the resected bowel. There was no sign of peritonitis and a primary anastomosis was performed. Intussusception was not found intraoperatively, the pathology seen on the CT scan was Meckel's diverticulum. Due to cardiorespiratory instability, the patient was ventilated for 4 days postoperatively, and he then recovered well from surgery.

**Fig. 2:**
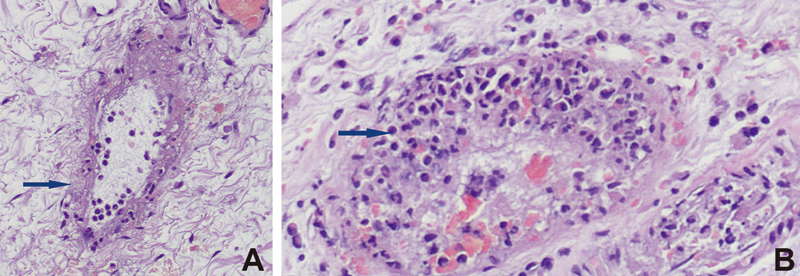
Necrosis of the vessel wall with neutrophilic infiltration was so severe in some areas that vascular structures were barely recognizable: Vasculitis in the intestinal submucosa and fibrinoid necrosis in the vascular wall can be seen ( **A** , hematoxylin and eosin staining, 100x zoom). Vasculitis seen in the submucosa of the gangrenous inflamed Meckel's diverticulum. Neutrophile granulocytes and fibrinoid necrosis deposits in the vascular wall ( **B** , hematoxylinand eosin staining, 100x zoom).

The histopathology examination revealed severe necrosis of the bowel. Severe vasculitis of the mesenteric vessels was also obvious. Necrosis of the vessel wall with neutrophilic infiltration was significant and severe in some areas that the vascular structures were barely recognizable ( Fig. 2A ). Similar features were seen on the diverticulum ( Fig. 2B ).

## Discussion

The first patient with PIMS-related intestinal ischemia published by Khesrani et al had a complex history with medullar aplasia and received long-term steroid treatment. Our patient was not immunocompromised but had a history of developmental delay, left renal aplasia, and suboptimal social circumstances. This might have contributed to his initial severe dehydration.

Both children, our case reported here and the one from Khesrani et al, presented with similar symptoms such as high temperature and vomiting, hallucinations, or confusion and ecchymosis/petechiae. The inflammatory markers were similarly high CRP of 242 vs. 221 mg/L. In Keshrani et al'scase, the reverse transcriptase-polymerase chain reaction (RT-PCR) of the nasopharyngeal swab was positive. In our case, this test was negative; however, the antibody serology was positive, and the D-dimer, which is considered as a COVID-19 biomarker was extremely high too. This is more consistent with post-infection PIMS; however, in the first case by Khesrani et al, antibody testing was not reported.

The laparotomy findings were very similar in both cases. However, ileal necrosis in our case was more extensive. This may be influenced by the obstruction, i.e., the distension of the bowel wall caused by complicated Meckel's diverticulum.

There are a few other cases of MIS with intestinal complications that required surgery in the literature. Jackson et al reported a case of appendicitis with a fecolith and a short, 3 cm segment of “inflamed” ileum was noted and subsequently resected in a COVID-19-positive child. 7 Shan et al reported a case with more extensive inflammation affecting the longer ileocolic segment, which formed a 6 cm diameter mass. 8

The bowel wall in these cases was thickened.

It was concluded that MIS in the intestine may have led to circumferential wall thickening and luminal narrowing, like the chronic inflammatory bowel disease, and could lead to bowel obstruction.

However, in Khesrani's and our case, no bowel wall thickening was noted in the necrotic resected bowel segment. It seems in both of these cases, acute intestinal necrosis was rather a consequence of a vascular occlusion. It is possible that vasculitis led a rapid occlusion of the mesenteric vessels and was less extensive in the bowel wall.

In Keshrani's case, the histology demonstrated a hemorrhagic infarction, lesions with foci of ischemic necrosis without evidence of a thrombus, whereas in our case, vasculitis in the intestinal submucosa and fibrinoid necrosis in the vascular wall could be seen ( Fig. 2 ). Thrombosis of the mesenteric veins of the necrotic bowel was macroscopically obvious in our case ( Fig. 1B ).

One can argue the intestinal inflammation not related to COVID-19. This might be true for Meckel's diverticulitis, however would not explain the long ischemic small bowel. We hypothesize that the vasculitis affected first the vitello-intestinal artery, which is a thin and elongated vessel arising from the ileal branch of the superior mesenteric artery. 9 This led to the necrosis of Meckel's diverticulum with peritonitis and small bowel obstruction. The obstruction developed gradually and was not present on admission (normal bowel movements, no dilated bowel loops, and normal bowel sounds initially). However, the extent of the small bowel necrosis (100 cm) cannot be simply explained with the mechanical bowel obstruction caused by Meckel's diverticulitis alone. There was no further adhesion or band to blame. Extensive thrombosis of the mesentery (not a common feature in Meckel's diverticulitis) suggests that vasculitis and ischemia may have played a significant role in this case.

It is important to note that beyond the confirmed pneumonia, both patients had significant dehydration or even shock (in Keshrani et al'scase). 5 The combination of vasculitis, hypoxia, and hypotensive episodes may have contributed to the very similar outcome in both cases.

Despite no perforation was reported, ileostomy was performed in the first case; however, in our case, a primary anastomosis was successful. This is in line with the reports of aseptic peritonitis with PIMS. 4 5 10

In severe COVID-19 infections, activated neutrophilsare found to partially deplete their granules containing the extracellular webs of chromatin, antimicrobial proteins, and enzymes to contain the infection (neutrophil extracellular trap [NET] within the vessels. Intravascular aggregation of NETs leads to the rapid occlusion of the affected vessels resulting in ischemia. 11 This may have played a role in our case as in COVID-19 infection, high neutrophil ratio and vessel occlusion were clinically identified; however specific tests such as flow cytometry, enzyme-linked immunosorbent assays, and neutrophile elastase immunohistochemistry were not performed.

No corticosteroids, heparin, or thrombolysis were introduced as the pathology seemed to be localized on the mesentery of the ischemic bowel and that was resected; however,acetylsalicylic acid was used postoperatively.

PIMS is known to mimic acutely a surgical abdomen-like appendicitis; however, patients with PIMS can develop real surgical emergencies such as appendicitis, intussusception, and intestinal ischemia and necrosis. 6 7 10 Fortunately, COVID-19-related intestinal ischemia is rare. Early diagnosis remains difficult; however, clinicians should be aware of this possible complication. Routine color Doppler US should be considered in all cases with abdominal pain with positive COVID-19 PCR or antibody test. Medical management still remains the primary management. Routine medical-thrombosis prophylaxis may be worth to consider. Thrombolytic therapy may be used in selected cases. Repeated physical examinations and consultation with surgeons are mandatory. In case of clinical deterioration, bowel obstruction and peritonitis surgical exploration may be necessary.

## Notes

New Insights and the Importance for the Pediatric Surgeon Acute abdominal pain is a hard differential diagnostic problem in PIMS patients.
